# Complete genome sequence of *Kosakonia pseudosacchari* RX.G5M8, a putative methylotroph isolated from Hong Kong soil

**DOI:** 10.1128/MRA.00469-23

**Published:** 2023-09-29

**Authors:** D. Y. Xu, K. M. Leung, G. K. K. Lai, S. D. J. Griffin

**Affiliations:** 1 Shuyuan Molecular Biology Laboratory, The Independent Schools Foundation Academy, Hong Kong SAR, China; DOE Joint Genome Institute, Berkeley, California, USA

**Keywords:** methylotrophs, *Kosakonia pseudosacchari*, soil microbiology

## Abstract

*Kosakonia pseudosacchari* RX.G5M8, a putative methylotroph, was isolated from garden soil in Hong Kong. Its complete genome, a single chromosome of 4,953,935 bp (GC content 53.91%), was established through hybrid assembly.

## ANNOUNCEMENT

Genus *Kosakonia*, designated in 2013 after reassignment of four species of *Enterobacter* (1), currently comprises at least nine species (2). These include *K. sacchari*, first isolated from *Saccharum officinarum* (3, 4); and, *K. pseudosacchari*, found as a *Zea mays* endophyte (5). While *Kosakonia* spp. are rarely human pathogens, *K. quasisacchari* and *K. cowanii* have been isolated from sites of infection (6, 7). Nevertheless, the significant antimicrobial resistance and virulence genes of *K. cowanii* strain 888-76T (8) seem uncommon in the other species. Instead, these are more often recognized as plant growth-promoting rhizobacteria with plant-beneficial genes enabling phosphate solubilization, siderophore production, nitrogen fixation, as well as indole-3-acetic acid and ethylene synthesis (9–13). Here, RX.G5M8 was isolated from soil screened for methylotrophy, a plant-associated trait previously observed in *K. arachidis* (14).

A 0.1 g sample of garden soil collected in Hong Kong (22.3038099 N, 114.1616503 E; 7 November 2021) was vortexed with 900 µL 0.9% (wt/vol) saline and a 10-µL aliquot used to inoculate 1 mL of a minimal salts medium (MSM) (15) containing 5% (vol/vol) methanol as the only carbon source. Following incubation at 27°C for 7 days with shaking, the mixture was streaked onto Luria agar (16), giving cream-colored colonies after overnight growth. Selected colonies were passaged to purity on Luria agar and reassessed for growth in MSM containing 1% (vol/vol) methanol by plate counts of aliquots withdrawn daily over 5 days. For DNA extraction (Qiagen DNeasy PowerSoil Pro Kit), a single colony of RX.G5M8, which showed moderate growth in 1% methanol, was spread on Luria agar and incubated for 24 h before harvesting.

A paired-end short-read sequencing library (NEB Next Ultra DNA Library Prep Kit) was sequenced via the NovaSeq 6000 platform using an SP PE250 flowcell and v1.5 Reagent Kit. Reads were quality filtered and trimmed using TrimGalore! v0.6.7 (https://github.com/FelixKrueger/TrimGalore) (stringency: 3; -e: 0.2), giving 2,137,624 read pairs (mean length, 250  bp) totaling ~534 Mbp. Long-read libraries, prepared from the same extracted DNA using the Rapid Barcoding Kit SQK-RBK004 (without size selection), were sequenced via Oxford Nanopore’s Spot-ON Flow Cell (vR9.4.1) and MinION sequencer, with MinKNOW v20.06.2 software and basecalling by Guppy v6.1.5 high-accuracy mode. The final long-read data set, trimmed by Filtlong v0.2.1 (https://github.com/rrwick/Filtlong) (min_length: 2,000; target_bases: 500 Mbp; length_weight: 10), totaled 63,710 reads (984 Mbp) with mean length 15,448 bp (N50 19,020). Default parameters were used for all software unless otherwise specified.

Unicycler v0.4.3 (17) combined the Illumina and MinION data sets to yield a circular chromosome of 4,953,935 bp (53.91% G + C, mean coverage 199×), which was submitted to NCBI PGAP v5.0 (18) for annotation. JSpeciesWS (19) found RX.G5M8 closest to *Kosakonia pseudosacchari* strain BDA62-3 (CP063425), with an average nucleotide identity of 98.67%.

RX.G5M8 possesses a glutathione-dependent formaldehyde detoxification pathway for methanol metabolism (20). The nitrogen-fixing Nif-regulon is at locus PF050_12345 to PF050_12440, and the recently identified non-mobile colistin resistance enzyme (NMCR-2) (21) is encoded at locus PF050_10580.

## Data Availability

The complete genome sequence and raw sequence data for *Kosakonia pseudosacchari* RX.G5M8 are available through NCBI under BioProject PRJNA922486, with GenBank and SRA accession numbers CP115712, SRR24734531 (MinION), and SRR24734532 (NovaSeq), respectively.

## References

[1] Brady C, Cleenwerck I, Venter S, Coutinho T, De Vos P. 2013. Taxonomic evaluation of the genus Enterobacter based on multilocus sequence analysis (MLSA): proposal to reclassify E. nimipressuralis and E. amnigenus into Lelliottia gen. nov. as Lelliottia nimipressuralis comb. nov. and Lelliottia amnigena comb. nov., respectively, E. gergoviae and E. pyrinus into Pluralibacter gen. nov. as Pluralibacter gergoviae comb. nov. and Pluralibacter pyrinus comb. nov., respectively, E. cowanii, E. radicincitans, E. oryzae and E. arachidis into Kosakonia gen. nov. as Kosakonia cowanii comb. nov., Kosakonia radicincitans comb. nov., Kosakonia oryzae comb. nov. and Kosakonia arachidis comb. nov., respectively, and E. turicensis, E. helveticus and E. pulveris into Cronobacter as Cronobacter zurichensis nom. nov., Cronobacter helveticus comb. nov. and Cronobacter pulveris comb. nov., respectively, and emended description of the genera Enterobacter and Cronobacter. Syst Appl Microbiol 36:309–319. doi:10.1016/j.syapm.2013.03.005 23632228

[2] Gao G, Zhang Y, Niu S, Chen Y, Wang S, Anwar N, Chen S, Li G, Ma T. 2022. Reclassification of Enterobacter sp. FY-07 as Kosakonia oryzendophytica FY-07 and its potential to promote plant growth. Microorganisms 10:575. doi:10.3390/microorganisms10030575 35336150 PMC8951479

[3] Zhu B, Zhou Q, Lin L, Hu C, Shen P, Yang L, An Q, Xie G, Li Y. 2013. Enterobacter sacchari sp. nov., a nitrogen-fixing bacterium associated with sugar cane (Saccharum officinarum L.). Int J Syst Evol Microbiol 63:2577–2582. doi:10.1099/ijs.0.045500-0 23291881

[4] Chen M, Zhu B, Lin L, Yang L, Li Y, An Q. 2014. Complete genome sequence of Kosakonia sacchari type strain Sp1^T^. Stand Genomic Sci 9:1311–1318. doi:10.4056/sigs.5779977 25197499 PMC4149035

[5] Kämpfer P, McInroy JA, Doijad S, Chakraborty T, Glaeser SP. 2016. Kosakonia pseudosacchari sp. nov., an endophyte of Zea mays. Syst Appl Microbiol 39:1–7. doi:10.1016/j.syapm.2015.09.004 26597455

[6] Wang C, Wu W, Wei L, Feng Y, Kang M, Xie Y, Zong Z. 2019. Kosakonia quasisacchari sp. nov. recovered from human wound secretion in China. Int J Syst Evol Microbiol 69:3155–3160. doi:10.1099/ijsem.0.003606 31355737

[7] Berinson B, Bellon E, Christner M, Both A, Aepfelbacher M, Rohde H. 2020. Identification of Kosakonia cowanii as a rare cause of acute cholecystitis: case report and review of the literature. BMC Infect Dis 20:366. doi:10.1186/s12879-020-05084-6 32448208 PMC7245821

[8] Yang X-J, Wang S, Cao J-M, Hou J-H. 2018. Complete genome sequence of human pathogen Kosakonia cowanii type strain 888-76T. Braz J Microbiol 49:16–17. doi:10.1016/j.bjm.2017.03.010 28774637 PMC5790570

[9] Romano I, Ventorino V, Ambrosino P, Testa A, Chouyia FE, Pepe O. 2020. Development and application of low-cost and eco-sustainable bio-stimulant containing a new plant growth-promoting strain Kosakonia pseudosacchari Tl13. Front Microbiol 11:2044. doi:10.3389/fmicb.2020.02044 33013749 PMC7461993

[10] Quintas-Nunes F, Rossi MJ, Nascimento FX. 2022. Genomic insights into the plant-associated lifestyle of Kosakonia radicincitans MUSA4, a diazotrophic plant-growth-promoting bacterium. Syst Appl Microbiol 45:126303. doi:10.1016/j.syapm.2022.126303 35149280

[11] Singh RK, Singh P, Guo D-J, Sharma A, Li D-P, Li X, Verma KK, Malviya MK, Song X-P, Lakshmanan P, Yang L-T, Li Y-R. 2021. Root-derived endophytic diazotrophic bacteria Pantoea cypripedii Af1 and Kosakonia arachidis Ef1 promote nitrogen assimilation and growth in sugarcane. Front Microbiol 12:774707. doi:10.3389/fmicb.2021.774707 34975800 PMC8714890

[12] Meng X, Bertani I, Abbruscato P, Piffanelli P, Licastro D, Wang C, Venturi V. 2015. Draft genome sequence of rice endophyte-associated isolate Kosakonia oryzae Ko348. Genome Announc 3:e00594-15. doi:10.1128/genomeA.00594-15 26044436 PMC4457073

[13] Feng B-Y, Zhang M-T, Su G-X, Bao Y-Q, Xu Y, Chen Y-P. 2022. Siderophore synthesis ability of the nitrogen-fixing bacterium (NFB) GXGL-4A is regulated at the transcriptional level by a transcriptional factor (trX) and an aminomethyltransferase-encoding gene (amt). Curr Microbiol 79:369. doi:10.1007/s00284-022-03080-4 36253498

[14] Madhaiyan M, Poonguzhali S, Lee J-S, Saravanan VS, Lee K-C, Santhanakrishnan P. 2010. Enterobacter arachidis sp. nov., a plant-growth-promoting diazotrophic bacterium isolated from rhizosphere soil of groundnut. Int J Syst Evol Microbiol 60:1559–1564. doi:10.1099/ijs.0.013664-0 19684326

[15] Zhang C, Li C, Zhang L, Shao X. 2012. Isolation and characterization of a benzoic acid degrading Pseudomonas putida from a pharmaceutical wastewater treatment plant. Afr J Microbiol Res 6:4608–4613. doi:10.5897/AJMR12.548

[16] MacWilliams MP, Liao MK, American Society for Microbiology. 2006. Luria broth (LB) and Luria Agar (LA) media and their uses protocol. ASM MicrobeLibrary. Available from: https://asm.org/Protocols/Luria-Broth-LB-and-Luria-Agar-LA-Media-and-Their-U

[17] Wick RR, Judd LM, Gorrie CL, Holt KE. 2017. Unicycler: resolving bacterial genome assemblies from short and long sequencing reads. PLoS Comput Biol 13:e1005595. doi:10.1371/journal.pcbi.1005595 28594827 PMC5481147

[18] Haft DH, DiCuccio M, Badretdin A, Brover V, Chetvernin V, O’Neill K, Li W, Chitsaz F, Derbyshire MK, Gonzales NR, Gwadz M, Lu F, Marchler GH, Song JS, Thanki N, Yamashita RA, Zheng C, Thibaud-Nissen F, Geer LY, Marchler-Bauer A, Pruitt KD. 2018. RefSeq: an update on prokaryotic genome annotation and curation. Nucleic Acids Res 46:D851–D860. doi:10.1093/nar/gkx1068 29112715 PMC5753331

[19] Richter M, Rosselló-Móra R, Oliver Glöckner F, Peplies J. 2016. Jspeciesws: a web server for prokaryotic species circumscription based on pairwise genome comparison. Bioinformatics 32:929–931. doi:10.1093/bioinformatics/btv681 26576653 PMC5939971

[20] Gonzalez CF, Proudfoot M, Brown G, Korniyenko Y, Mori H, Savchenko AV, Yakunin AF. 2006. Molecular basis of formaldehyde detoxification. characterization of two S-formylglutathione hydrolases from Escherichia coli, FrmB and YeiG. J Biol Chem 281:14514–14522. doi:10.1074/jbc.M600996200 16567800

[21] Ullah S, Ji K, Li J, Xu Y, Jiang C, Zhang H, Huang M, Feng Y. 2021. Characterization of NMCR-2, a new non-mobile colistin resistance enzyme: implications for an MCR-8 ancestor. Environ Microbiol 23:844–860. doi:10.1111/1462-2920.15171 32686285

